# Phase III, randomized, double-blind, placebo-controlled, multicenter study of lipegfilgrastim in patients with non-small cell lung cancer receiving myelosuppressive therapy

**DOI:** 10.1186/s40064-015-1067-7

**Published:** 2015-07-03

**Authors:** Constantin Volovat, Igor M Bondarenko, Oleg A Gladkov, Reiner Elsässer, Anton Buchner, Peter Bias, Udo Müller

**Affiliations:** Centrul de Oncologie Medicala, Vasile Conta 2 Str, 700106 Iasi, Romania; Dnipropetrovsk State Medical Academy, City Clinical Hospital, N 4, 31, Glizhnaya Str, Dnipropetrovsk, 49102 Ukraine; Chelyabinsk Regional Clinical Oncology Dispensary, 42, Blukhera Str., Chelyabinsk, 454087 Russia; Teva Ratiopharm, Merckle GmbH, Graf-Arco-Strasse 3, 89079 Ulm, Germany; Teva Pharmaceuticals, Inc., Graf-Arco-Strasse 3, 89079 Ulm, Germany

**Keywords:** Neutropenia, Non-small cell lung cancer, Recombinant granulocyte colony-stimulating factor, Lipegfilgrastim, Phase III clinical trial

## Abstract

**Purpose:**

The aim of this study was to demonstrate lipegfilgrastim superiority versus placebo in adults with non-small cell lung cancer receiving myelosuppressive chemotherapy.

**Methods:**

This phase III, double-blind study randomized chemotherapy-naive patients to receive cisplatin and etoposide with either lipegfilgrastim 6 mg or placebo. Because of the placebo control, patients at individual high risk for febrile neutropenia (FN; ≥20%) were excluded. Study drug was administered on day 4 (24 h after chemotherapy) of a 21-day cycle for ≤4 cycles. Primary efficacy measure was FN incidence in cycle 1. Secondary assessments included duration of severe neutropenia (DSN), absolute neutrophil count (ANC) profile, and adverse events (AEs).

**Results:**

The study included 375 patients (lipegfilgrastim, *n* = 250; placebo, *n* = 125). Lipegfilgrastim superiority for FN incidence in cycle 1 was not achieved but incidence was lower (2.4%) versus placebo (5.6%). Cycle 1 mean DSN was significantly shorter for lipegfilgrastim (0.6 ± 1.1 days) versus placebo (2.3 ± 0.5 days; *p* < 0.0001). Incidence of severe neutropenia was significantly lower for lipegfilgrastim versus placebo overall and in each cycle (all, *p* < 0.0001). Mean ANC nadir was lowest in cycle 1 but significantly higher for lipegfilgrastim (1.60 ± 1.64) than placebo (0.67 ± 0.85; *p* < 0.0001). Mean time to ANC recovery was shorter with lipegfilgrastim in each cycle. Treatment-emergent AEs were similar between treatment groups.

**Conclusions:**

Lipegfilgrastim was not statistically superior to placebo for incidence of FN in cycle 1, but was more effective in reducing incidence of severe neutropenia, DSN, and time to ANC recovery, with an acceptable safety profile.

Controlled-trials.com identifier: ISRCTN55761467.

## Background

Neutropenia is a major dose-limiting toxicity in many myelosuppressive chemotherapy regimens (Holmes et al. 2002; Crawford et al. 2013). A patient’s risk of developing neutropenia or febrile neutropenia (FN) depends on several factors, including the type of cancer, chemotherapy regimen (standard-dose, dose-dense, or high-dose therapy), and patient-related and disease-related factors, such as age and comorbidities (Crawford et al. 2013).

Recombinant granulocyte colony-stimulating factors (G-CSFs) promote the proliferation and differentiation of neutrophils, alleviating the severity of chemotherapy-induced neutropenia and FN (Cooper et al. 2011). These agents are well established as primary prophylaxis for FN and are recommended in European and US guidelines for chemotherapy patients at high (≥20%) risk of FN (Crawford et al. 2010, 2013; Smith et al. 2006; Aapro et al. 2011). Filgrastim, the first recombinant human G-CSF, requires daily subcutaneous (SC) injections (Neupogen [package insert] 2013). With the attachment of polyethylene glycol (PEG) to the G-CSF molecule, the half-life of pegfilgrastim was extended compared with filgrastim, allowing once-per-chemotherapy cycle administration (Neulasta [package insert] 2012). Lipegfilgrastim is a glycoPEGylated, once-per-cycle recombinant human G-CSF expressed in *Escherichia coli.* It is approved by the European Medicines Agency for reducing the duration of neutropenia and the incidence of FN in adults treated with cytotoxic chemotherapy for malignancy (with the exception of chronic myeloid leukemia and myelodysplastic syndromes) (Lonquex 2013).

A recent phase III trial (controlled-trials.com identifier ISRCTN56891934) demonstrated the clinical efficacy of lipegfilgrastim to be noninferior to pegfilgrastim in reducing neutropenia in breast cancer patients receiving myelosuppressive chemotherapy (Bondarenko et al. 2013). This trial was conducted to demonstrate superiority of once-per-cycle lipegfilgrastim versus placebo in patients with stage IIIb/IV non-small cell lung cancer (NSCLC) receiving up to four cycles of cisplatin and etoposide chemotherapy. Evaluations of efficacy, tolerability, and pharmacokinetic properties were secondary assessments.

## Results

**Figure 1:**
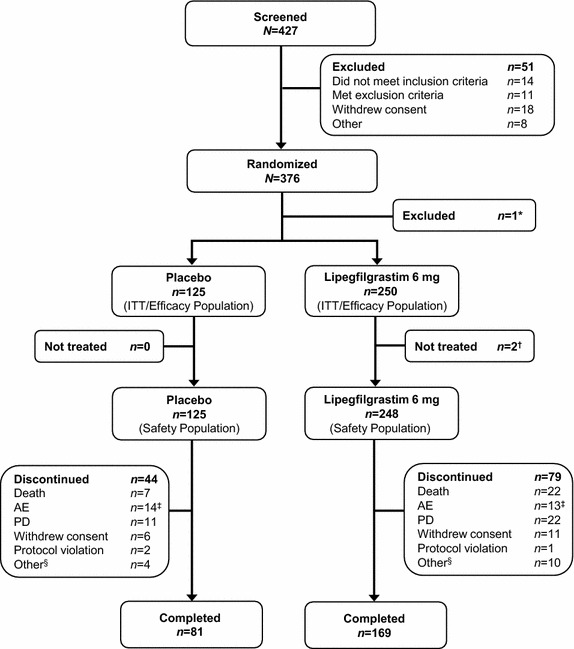
Patient disposition from randomization to study completion. *One patient randomized in error received no chemotherapy and no study medication and was excluded from all statistical analyses and populations. ^†^Two patients who received chemotherapy but died after randomization, before study medication was administered, were included in the efficacy population but not in the safety population. ^‡^Adverse events listed as the primary reason for study discontinuation include placebo patients: two patients each with febrile neutropenia, cerebral infarction, and pneumonia; one patient each with back pain, general physical health deterioration, arterial thrombosis in a limb, pain in the extremities, inadequate control of diabetes mellitus, tumor lysis syndrome, and neutropenia; and one patient with anemia, thrombocytopenia, and neutropenia; lipegfilgrastim patients: two patients with anemia; one patient each with wound necrosis, syphilis, atrial fibrillation, pyothorax, fatigue, increased aspartate aminotransferase, gastric hemorrhage, dementia, pulmonary embolism, asthenia, and hemoptysis. ^§^Includes patients lost to follow-up (*n* = 2), treatment failure (*n* = 3), and other (*n* = 5). *AE* adverse event, *ITT* intent to treat, *PD* progression of underlying disease.

The study was conducted between May 2010 and April 2011 at 72 centers in eight countries. In total, 427 patients were screened and 376 were randomized (Belarus, *n* = 34; Bosnia-Herzegovina, *n* = 2; Bulgaria, *n* = 16; Poland, *n* = 4; Romania, *n* = 25; Russia, *n* = 160; Serbia, *n* = 20; Ukraine, *n* = 115). One patient in the lipegfilgrastim group who was randomized in error and received no chemotherapy or study medication was excluded from the efficacy and safety analyses. Thus, 250 patients in the lipegfilgrastim group and 125 in the placebo group were included in the intent-to-treat population (Figure 1). Of these, 169 (67.6%) patients in the lipegfilgrastim and 81 (64.8%) in the placebo group completed treatment. Two patients in the lipegfilgrastim group who died after randomization but did not receive study medication were included in the efficacy but not safety analyses.

### Patients

Baseline demographics and clinical characteristics were similar between treatment groups (Table 1). Most patients were men, had stage IV NSCLC, an Eastern Cooperative Oncology Group Performance Status (ECOG PS) of 1, and were receiving chemotherapy for metastatic disease. The mean age was similar in both groups and was somewhat younger than is typically seen for patients with lung cancer. However, this was expected as one of the risk factors that would have contributed to the exclusion of patients at individual high risk for FN was age >65 years, leading to a relatively low percentage of patients aged >65 years (23.5%) being enrolled.

**Table 1:** Patient demographics and baseline characteristics (intent-to-treat population)

Characteristic	Placebo (*n* = 125)	Lipegfilgrastim 6 mg SC (*n* = 250)
Age, years		
Mean ± SD	58.7 ± 8.5	58.2 ± 8.5
≤64, *n* (%)	94 (75.2)	193 (77.2)
65–74, *n* (%)	29 (23.2)	54 (21.6)
≥75, *n* (%)	2 (1.6)	3 (1.2)
Weight, kg		
Mean ± SD	70.4 ± 13.4	69.0 ± 12.9
≤60, *n* (%)	34 (27.2)	70 (28.0)
>60 to ≤75, *n* (%)	53 (42.4)	106 (42.4)
>75, *n* (%)	38 (30.4)	74 (29.6)
Sex, n (%)		
Female	20 (16.0)	30 (12.0)
Male	105 (84.0)	220 (88.0)
Region, *n* (%)		
Russia	54 (43.2)	106 (42.4)
Ukraine	38 (30.4)	77 (30.8)
Rest of Europe	33 (26.4)	67 (26.8)
NSCLC stage at enrolment, *n* (%)		
Stage IIIB	49 (39.2)	97 (38.8)
Stage IV	76 (60.8)	152 (60.8)
Unknown	0 (0)	1 (0.4)
Time since diagnosis, months		
Mean ± SD	3.4 ± 9.1	2.4 ± 6.2
Median (range)	1.0 (0–58.0)	1.0 (0–52.0)
ECOG PS, *n* (%)		
0	19 (15.2)	28 (11.2)
1	96 (76.8)	194 (77.6)
2	10 (8.0)	28 (11.2)
Reason for chemotherapy, *n* (%)		
Adjuvant therapy	21 (16.8)	35 (14.0)
Treatment for metastatic disease	104 (83.2)	215 (86.0)
Lung cancer surgery		
No	98 (78.4)	215 (86.0)
Yes	27 (21.6)	35 (14.0)

*ECOG PS* Eastern Cooperative Oncology Group Performance Status, *NSCLC* non-small cell lung cancer, *SC* subcutaneously, *SD* standard deviation.

Most patients in both treatment groups received their planned chemotherapy dose in each cycle; ≤3.3% of patients receiving placebo and ≤2.3% of those receiving lipegfilgrastim had a dose reduction or omission. The total number of administered doses for both cisplatin and etoposide was similar between treatment groups (data not shown). The proportion of patients with chemotherapy dose delays was significantly lower in patients treated with lipegfilgrastim than in those receiving placebo (cycle 2, 28.5 vs. 65.1%; cycle 3, 42.1 vs. 66.3%; and cycle 4, 40.4 vs. 75.3%, respectively; all *p* ≤ 0.0001). The mean duration of chemotherapy delay across all cycles also was shorter for patients receiving lipegfilgrastim (6.4 ± 7.6 days) versus those receiving placebo (12.8 ± 10.2 days).

### Efficacy

The primary efficacy measure, incidence of FN in cycle 1, was lower in the lipegfilgrastim group compared with the placebo group (Table 2), but the difference was not significant. Thus, the study failed to achieve its primary objective of demonstrating superiority of lipegfilgrastim versus placebo in these patients.

**Table 2:** Febrile neutropenia in cycles 1, 2, 3, and 4 (intent-to-treat population)

Cycle	Placebo			Lipegfilgrastim 6 mg SC			Lipegfilgrastim 6 mg SC vs. placebo		
	*N*	FN	%	*N*	FN	%	OR	95% CI	*P* value*
1	125	7	5.6	250	6	2.4	0.390	0.121–1.260	0.1151
2	105	0	0	214	1	0.5	NE	NE	0.9551
3	92	1	1.1	188	1	0.5	0.642	0.234–1.762	0.3883
4	81	2	2.5	171	2	1.2	0.421	0.119–1.489	0.1787

*CI* confidence interval, *FN* febrile neutropenia, *NE* not evaluable, *OR* odds ratio, *SC* subcutaneously.

* *P* values based on a null hypothesis of odds ratio = 1.

Seven investigator-assessed cases of FN were observed during cycles 2, 3, and 4 (Table 2), but no significant differences in the incidence of FN between treatment groups were observed (*p* > 0.05). None of the patients switched to open-label lipegfilgrastim experienced FN during chemotherapy cycles 2, 3, and 4. Patients receiving lipegfilgrastim experienced a significantly shorter duration of severe neutropenia (DSN) in cycle 1 compared with patients receiving placebo (*p* < 0.0001; Table 3). Similarly, the DSN in cycles 2, 3, and 4 was consistently and significantly shorter in the lipegfilgrastim group compared with the placebo group (*p* < 0.0001, all cycles). The incidence of severe neutropenia was significantly lower in the lipegfilgrastim group versus the placebo group overall (*p* < 0.0001) and in each cycle (*p* < 0.0001, each cycle; Table 4). Notably, in cycles 2, 3, and 4, approximately 80% of patients in the lipegfilgrastim group experienced no severe neutropenia compared with approximately 40% in the placebo group.

**Table 3:** Duration of severe neutropenia in cycles 1, 2, 3, and 4 (ITT population)

Cycle	Duration of severe neutropenia (days)	Placebo	Lipegfilgrastim 6 mg SC
1	*N*	125	250
	Mean ± SD	2.3 ± 2.5	0.6 ± 1.1
	Median (range)	2.0 (0–11.0)	0 (0–5.0)
	LSM*	−1.661	
	95% CI*	−2.089 to −1.232	
	*P* value*	<0.0001	
2	*N*	122	244
	Mean ± SD	2.2 ± 2.6	0.3 ± 0.7
	Median (range)	1.0 (0–11.0)	0 (0–4.0)
	LSM*	−1.915	
	95% CI*	−2.317 to −1.512	
	*P* value*	<0.0001	
3	*N*	122	245
	Mean ± SD	2.0 ± 2.4	0.4 ± 0.9
	Median (range)	1.0 (0–11.0)	0 (0–5.0)
	LSM*	−1.640	
	95% CI*	−2.053 to −1.227	
	*P* value*	<0.0001	
4	*N*	123	246
	Mean ± SD	2.3 ± 2.5	0.5 ± 1.1
	Median (range)	1.0 (0–11.0)	0 (0–0.8)
	LSM*	−1.844	
	95% CI*	−2.281 to −1.407	
	*P* value*	<0.0001	

Includes patients from the ITT population who were withdrawn from the study.

*CI* confidence interval, *ITT* intent to treat, *LSM* least squares mean, *SC* subcutaneously, *SD* standard deviation.

*Least squares mean, 95% CI, and *P* value are for Poisson regression analysis lipegfilgrastim–placebo.

**Table 4:** Incidence of severe and very severe neutropenia in cycles 1, 2, 3, and 4 (ITT population)

Cycle	Placebo			Lipegfilgrastim 6 mg SC			Lipegfilgrastim 6 mg SC vs. placebo		
	*N*	*n*	%	*N*	*n*	%	Odds ratio	95% CI	*P* value*
Severe neutropenia (grade 4, ANC <0.5 × 10^9^/L)									
1	125	74	59.2	249	80	32.1	0.325	0.206–0.512	<0.0001
2	105	55	52.4	215	36	16.7	0.156	0.086–0.282	<0.0001
3	92	47	51.1	188	26	13.8	0.115	0.057–0.229	<0.0001
4	81	45	55.6	169	25	14.8	0.121	0.062–0.238	<0.0001
All	125	100	80.0	249	103	41.4	0.176	0.105–0.294	<0.0001
Very severe neutropenia (ANC <0.1 × 10^9^/L)									
1	125	18	14.4	249	27	10.8	0.700	0.365–1.342	NS
2	105	10	9.5	215	8	3.7	0.298	0.099–0.895	0.031
3	92	9	9.8	188	9	4.8	0.421	0.156–1.138	NS
4	81	11	13.6	169	8	4.7	0.260	0.098–0.687	0.007
All	125	33	26.4	249	40	16.1	0.516	0.300–0.888	0.017

Based on data actually collected.

*ANC* absolute neutrophil count, *CI* confidence interval, *ITT* intent to treat, *NS* not significant, *SC* subcutaneously.

*P values based on a null hypothesis of odds ratio = 1.

Patients treated with lipegfilgrastim experienced a shorter mean duration of very severe neutropenia [absolute neutrophil count (ANC) <0.1 × 10^9^/L] versus patients receiving placebo in cycle 1: 0.3 ± 0.9 days versus 0.2 ± 0.6 days for lipegfilgrastim and placebo, respectively. The mean duration of very severe neutropenia was also shorter for patients in the lipegfilgrastim group compared with the placebo group in cycles 2, 3, and 4. Patients receiving lipegfilgrastim had a significantly lower incidence of very severe neutropenia over all cycles versus those receiving placebo (*p* = 0.017). Differences between groups also were significant in cycles 2 (*p* = 0.031) and 4 (*p* = 0.007), with the lipegfilgrastim group having a lower incidence (Table 4).

**Figure 2:**
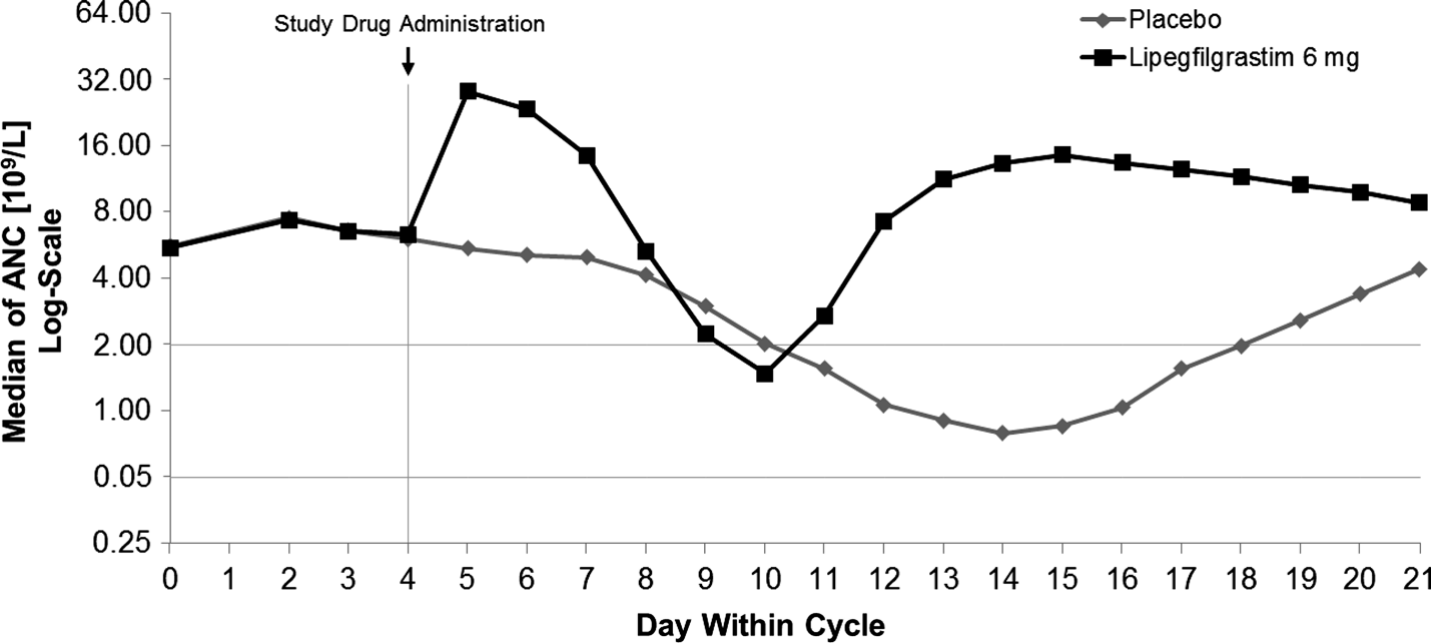
Time course of measured median absolute neutrophil count in cycle 1 (intent-to-treat population). *ANC* absolute neutrophil count.

The time course of median ANC during cycle 1 is shown in Figure 2. The mean depth of the ANC nadir in both treatment groups was lowest in cycle 1, but was significantly higher in patients treated with lipegfilgrastim (1.6 ± 1.6 × 10^9^/L) versus patients receiving placebo (0.7 ± 0.9 × 10^9^/L); *p* < 0.0001). In cycles 2, 3, and 4, the mean ANC nadir was greater than 2.5 × 10^9^/L for the lipegfilgrastim group, but remained below 1.0 × 10^9^/L for the placebo group (mean 2.8 vs. 0.8, 2.8 vs. 0.8, and 2.6 vs. 0.7 × 10^9^/L, respectively; *p* < 0.0001 in each case).

The mean time to ANC nadir was consistently shorter in the lipegfilgrastim group compared with the placebo group in each cycle (cycle 1: 8.2 vs. 13.7; cycle 2: 9.6 vs. 13.6; cycle 3: 9.8 vs. 13.6; cycle 4: 9.6 vs. 14.2 days). In addition, the mean time to ANC recovery from ANC nadir was significantly shorter in the lipegfilgrastim versus placebo group in each cycle (cycle 1: 1.1 vs. 2.7; cycle 2: 0.6 vs. 2.6; cycle 3: 0.8 vs. 2.3; cycle 4: 0.7 vs. 2.6 days; *p* < 0.0001 between groups in each cycle). Patients treated with lipegfilgrastim also experienced a significantly shorter mean time to ANC recovery after chemotherapy in each chemotherapy cycle compared with patients receiving placebo (cycle 1: 6.8 vs. 13.0; cycle 2: 5.6 vs. 13.8; cycle 3: 6.0 vs. 13.7; cycle 4: 5.4 vs. 14.0 days, respectively; *p* < 0.0001 between groups in each cycle).

### Safety

During the double-blind phase, 171 (69.0%) patients in the lipegfilgrastim group and 81 (64.8%) in the placebo group received four doses of study medication. The mean total amount of study medication administered was 19.9 ± 6.7 mg and 19.3 ± 7.0 mg in the lipegfilgrastim and placebo groups, respectively.

Adverse events (AEs) were similar between treatment groups. The most common AEs (total incidence of ≥10% in either treatment group) in the lipegfilgrastim and placebo groups were alopecia (40.7 and 33.6%), anemia (25.4 and 24.0%), nausea (23.8 and 21.6%), neutropenia (20.6 and 35.2%), thrombocytopenia (12.9 and 8.0%), asthenia (11.3 and 18.4%), vomiting (11.3 and 12.0%), and leukopenia (6.5 and 11.2%), respectively (Table 5). In total, 57 (23.0%) patients in the lipegfilgrastim group and 33 (26.4%) patients in the placebo group experienced an AE that lead to discontinuation from the study.

**Table 5:** Most frequent TEAEs (≥5% of patients) in either treatment group across all cycles (safety population)

AE by preferred term	Placebo (*N* = 125)		Lipegfilgrastim 6 mg SC (*N* = 248)	
	*n*	*%*	*n*	*%*
Alopecia	42	33.6	101	40.7
Anemia	30	24.0	63	25.4
Nausea	27	21.6	59	23.8
Neutropenia	44	35.2	51	20.6
Thrombocytopenia	10	8.0	32	12.9
Asthenia	23	18.4	28	11.3
Vomiting	15	12.0	28	11.3
Decreased appetite	12	9.6	23	9.3
Hypokalemia	3	2.4	20	8.1
Leukopenia	14	11.2	16	6.5
Fatigue	6	4.8	16	6.5
Disease progression	5	4.0	16	6.5
NSCLC	4	3.2	16	6.5
Chest pain	8	6.4	14	5.6
Febrile neutropenia	10	8.0	11	4.4
Dyspnea	9	7.2	11	4.4

TEAEs include all AEs except those specifically rated by investigators as unrelated to study drug. Multiple mentions per patient are possible. TEAEs with onset after the start of prophylactic open-label lipegfilgrastim treatment are excluded.

*AE* adverse event, *NSCLC* non-small cell lung cancer, *SC* subcutaneously, *TEAEs* treatment-emergent adverse events.

Bone-pain-related symptoms (defined as arthralgia, back pain, bone pain, myalgia, noncardiac chest pain, and pain in extremity) were reported in 21 (8.5%) patients treated with lipegfilgrastim and 8 (6.4%) patients receiving placebo. These events were generally mild or moderate in severity and led to study discontinuation in only two patients (both receiving placebo).

Serious AEs in the lipegfilgrastim and placebo groups included anemia (3.2 and 1.6%), NSCLC (3.2 and 0.8%), disease progression (2.4 and 0%), FN (2.0 and 4.0%), neutropenia (1.6 and 0.8%), pulmonary embolism (1.2 and 1.6%), cardio-respiratory arrest (1.2% and 0), thrombocytopenia (1.2% and 0), pneumonia (0.8 and 2.4%), pulmonary hemorrhage (0.8% and 0), renal failure (0.8% and 0), and sudden death (0.8% and 0), respectively.

A total of 31 (12.5%) patients treated with lipegfilgrastim and nine (7.2%) patients receiving placebo died during the course of the study or up to 30 days after the last study drug injection. General disorders and administration site conditions accounted for eight deaths in the lipegfilgrastim group and two in the placebo group; benign, malignant, and unspecified neoplasms for eight in the lipegfilgrastim group and two in the placebo group; respiratory, thoracic, and mediastinal disorders for five in the lipegfilgrastim group and three in the placebo group; cardiac disorders for four in the lipegfilgrastim group and one in the placebo group; vascular disorders for two in the lipegfilgrastim group; renal and urinary disorders for two in the lipegfilgrastim group; nervous system disorders for one in the lipegfilgrastim group and one in the placebo group; and metabolism and nutrition disorders for one in the lipegfilgrastim group.

Changes over time in laboratory assessments were consistent with the underlying disease and with the chemotherapy treatment received and, in the lipegfilgrastim group, with G-CSF therapy.

## Discussion

In this study, patients with NSCLC and a low individual risk of FN who received lipegfilgrastim had a lower incidence of FN during cycle 1 (2.4%) compared with patients receiving placebo (5.6%). The incidence of FN was in line with previously published results for pegfilgrastim (Vogel et al. 2005). Because the difference between treatment groups was not statistically significant, the study did not achieve its primary efficacy measure of demonstrating the superiority of lipegfilgrastim versus placebo. Nevertheless, the reduction in the incidence of FN in cycle 1 of >50% in the lipegfilgrastim group versus the placebo group is clinically important, particularly when considering the potential serious effects of FN on the overall health of patients. The actual incidence of FN was lower than anticipated for the placebo group and higher than anticipated for the lipegfilgrastim group, affecting the statistical power of the study. The use of prophylactic G-CSFs is not recommended by treatment guidelines unless a patient’s risk for developing FN is high (≥20%); however, the current study used chemotherapy with a reported incidence of FN <20% and excluded patients with an individual high risk of developing FN (≥20% risk). Thus, the use of prophylactic G-CSF therapy in this study was experimental in nature and provides additional evidence for the recommendation that prophylactic G-CSFs should not be used in patients with a risk of FN <20% (Crawford et al. 2010, 2013; Smith et al. 2006; Aapro et al. 2011). The use of cisplatin and etoposide, along with the strict definition of FN, may have contributed to the overall rate of FN being lower than the rate reported for patients receiving placebo in previously published lung cancer studies using the same chemotherapy regimen (Bonomi et al. 2000; Cardenal et al. 1999; Eckardt et al. 2006; Hanna et al. 2006). In particular, excluding patients thought to be at high risk for FN led to a relatively low percentage of patients aged ≥65 years (24.8%). In a similar study of patients with NSCLC receiving myelosuppressive chemotherapy and adjuvant filgrastim therapy or placebo, 41.1% of all patients were aged ≥65 years. Of these patients, 71 and 43% experienced FN in the placebo and filgrastim groups, respectively (Crawford et al. 2005).

The study design and statistical assumptions of this phase III trial may have contributed to not achieving the primary measure of demonstrating superiority to placebo in the incidence of FN in cycle 1. The existence of effective G-CSF supportive care and the uniformity of treatment guidelines for the use of G-CSFs in patients at risk for developing chemotherapy-induced neutropenia mean that a placebo-controlled trial in this setting is now uncommon. In addition, the strict definition of FN used in this study may have excluded patients who otherwise may have been reported as having FN. Moreover, the incidence of FN in cycle 1 was used as the primary measure, whereas G-CSF studies often use DSN in cycle 1 as the primary measure.

At the time the study was conducted, cisplatin and etoposide chemotherapy at the doses used was recommended for patients with stage IIIb/IV NSCLC (Belani et al. 2005). The reported incidence of FN for this regimen ranged from 8 to 12% in a population with an average risk of FN (Bonomi et al. 2000; Cardenal et al. 1999; Eckardt et al. 2006; Hanna et al. 2006), permitting the placebo-controlled study design. After the completion of this study, a systematic review and meta-analysis of the use of G-CSFs for FN prophylaxis following chemotherapy was published (Cooper et al. 2011). Although a broad range of studies was included, no study of patients with NSCLC receiving cisplatin and etoposide chemotherapy was included. The meta-analysis reported a risk ratio for FN of 0.30 [95% confidence interval (CI), 0.14–0.65] for pegfilgrastim and 0.57 (95% CI, 0.48–0.69) for filgrastim. In the current study, the odds ratio (OR) for FN in cycle 1 of 0.39 (95% CI, 0.121–1.260) for the comparison of lipegfilgrastim with placebo is in line with these results.

Secondary objectives of this study included the duration and incidence of severe neutropenia, and ANC profile (including depth of and time to ANC nadir, and time to ANC recovery). For most of these secondary measures, treatment with lipegfilgrastim was superior to placebo. In each of the four chemotherapy cycles, DSN was significantly shorter in the lipegfilgrastim group versus the placebo group (*p* < 0.0001), and in cycles 2, 3, and 4; approximately 80% of the lipegfilgrastim group experienced no severe neutropenia compared with approximately 40% of the placebo group. The incidence of severe neutropenia also was significantly lower in the lipegfilgrastim group versus the placebo group (*p* < 0.0001). A commonly used primary measure in studies with G-CSFs, DSN in cycle 1 was used as a primary measure in a recently published study that found lipegfilgrastim to be noninferior to pegfilgrastim in patients with breast cancer (Bondarenko et al. 2013). In the current study, the depth of ANC nadir was significantly lower in patients treated with lipegfilgrastim versus patients receiving placebo (*p* < 0.0001), and time to ANC nadir was shorter with lipegfilgrastim treatment. Time to ANC recovery also was shorter in the lipegfilgrastim group compared with the placebo group. Furthermore, significantly fewer patients receiving lipegfilgrastim had chemotherapy dose delays compared with those receiving placebo. These results add further support to data indicating the clinical benefits of lipegfilgrastim (Bondarenko et al. 2013; Buchner et al. 2011).

The high incidence of AEs was anticipated in this population of patients with stage IIIb/IV NSCLC receiving chemotherapy, and AEs were consistent with the underlying disease and chemotherapy regimen administered. In addition to neutropenia, the most common AEs (≥10% in either group) were alopecia, anemia, nausea, thrombocytopenia, asthenia, vomiting, and leukopenia—all known to be associated with the chemotherapy agents used in this study. The incidence of study medication-related AEs was relatively low. Bone-pain-related symptoms, known AEs associated with G-CSF therapy, were generally mild or moderate in severity. The incidence of mortality in this study was low, considering that the study population had advanced-stage disease. Most deaths were related to NSCLC or to other underlying conditions and were not considered to be related to lipegfilgrastim treatment. Deaths occurred early in and did not increase during the study; no difference in mortality was observed at the end of the 12-month follow-up, suggesting that the difference in mortality between the lipegfilgrastim and placebo groups at the end of the study was likely a chance effect.

The incidence of FN in patients with NSCLC receiving lipegfilgrastim or placebo in this study is similar to those reported in two placebo-controlled studies of pegfilgrastim in patients with advanced or metastatic colorectal cancer receiving chemotherapy regimens reported to have a low risk of FN (allowing the use of a placebo group). Again, the use of prophylactic G-CSF therapy in this setting is experimental, as the main chemotherapy regimens administered (oxaliplatin, fluorouracil, and leucovorin [FOLFOX4] or irinotecan, fluorouracil [infusion], and leucovorin [FOLFIRI]) have been reported to have a 6 and 9% incidence of FN, respectively, in patients with advanced colorectal cancer (Smith et al. 2006). In the large phase III trial (*N* = 845), grade 3/4 FN was reported in 2.4% of patients receiving pegfilgrastim and in 5.7% of patients receiving placebo after four cycles of chemotherapy (*p* = 0.014) (Pinter et al. 2013). In the phase II study (*n* = 252), grade 3/4 FN was reported in 2% of pegfilgrastim recipients and 8% of placebo recipients after four cycles of chemotherapy (*p* = 0.04) (Hecht et al. 2010).

In this study in patients with stage IIIb/IV NSCLC, treatment with lipegfilgrastim did not demonstrate superiority to placebo in terms of the incidence of FN in cycle 1. Use of a G-CSF in this setting was investigational in that it was administered to patients contrary to current treatment guidelines (Crawford et al. 2010, 2013; Smith et al. 2006; Aapro et al. 2011), yet treatment with lipegfilgrastim was consistently more effective than placebo in reducing the duration and incidence of severe neutropenia and time to ANC recovery, with an acceptable safety and tolerability profile in this patient population.

## Methods

This phase III, multinational, multicenter, double-blind, randomized, placebo-controlled study (controlled-trials.com identifier ISRCTN55761467) was designed to demonstrate superiority of a fixed 6-mg dose of lipegfilgrastim (XM22, Lonquex; Teva Pharmaceuticals Ltd, Petach Tikva, Israel) versus placebo in patients with NSCLC. The study was conducted in eight European countries (Belarus, Bosnia-Herzegovina, Bulgaria, Poland, Romania, Russia, Serbia, Ukraine) and included four phases: screening and randomization, double-blind treatment (chemotherapy cycles 1–4), end-of-study (or withdrawal) visit, and antibody follow-up. The study design followed the European Medicines Agency’s guidelines for a confirmatory study (EMEA 2007). Everyone involved in the conduct of the study was blinded to study medications.

### Patients

Eligible patients were men and women aged ≥18 years of any ethnic origin with a diagnosis of stage IIIb/IV NSCLC, documented histologically or cytologically, and a life expectancy of at least 4 months. Patients had to be chemotherapy naive, eligible to receive four cycles of cisplatin and etoposide as myelosuppressive chemotherapy (i.e., baseline ANC ≥1.5 × 10^9^/L and platelets ≥100 × 10^9^/L), have an ECOG PS ≤2, and have adequate hepatic, cardiac, and renal function. Women of childbearing potential had to use an effective method of contraception.

Because this was a placebo-controlled study, patients with an individual high risk of developing FN (i.e., ≥20%) with regard to the cisplatin and etoposide chemotherapy were excluded from the study. Potential individual high risk factors were patient age >65 years, low ECOG PS, poor nutritional status, and liver, kidney, or cardiovascular disease. Risk factors were considered together to determine a patient’s risk of developing FN, and therefore having only one risk factor did not automatically result in exclusion from the study. Other exclusion criteria included previous exposure to any G-CSF within 6 months before randomization, treatment with systemically active antibiotics within 72 h before chemotherapy, chronic use of oral corticosteroids, prior radiation therapy within 4 weeks of randomization, prior bone marrow or stem cell transplantation, or concomitant malignancy (other than in situ melanoma, skin cancer, or cervical carcinoma) within the preceding 5 years. Women who were pregnant or breastfeeding were excluded.

### Study design and treatment

All patients received chemotherapy with cisplatin and etoposide and were randomized (2:1) to receive lipegfilgrastim or placebo. Randomization was performed by Biostatistics Merckle GmbH through an interactive voice response system, using a block size of 2 and stratified by country. Patients received up to four 21-day chemotherapy cycles. On day 1 of each cycle, patients received cisplatin 80 mg/m^2^ intravenously (IV), with etoposide 120 mg/m^2^ IV administered on days 1, 2, and 3. Patients had to have an ANC ≥1.5 × 10^9^/L and platelet count of ≥100 × 10^9^/L to begin the next full-dose cycle. A dose delay of up to 2 weeks was acceptable. Absolute neutrophil counts were determined at local or regional laboratories rather than a central laboratory, for logistical reasons; all other laboratory measures were determined by two central laboratories. In addition, the patient’s overall condition had to allow further chemotherapy treatment as determined by the treating investigator. Generally, any chemotherapy-related toxicity had to have resolved to at least grade 1 toxicity prior to continuation of chemotherapy.

Patients received one dose of lipegfilgrastim 6 mg or placebo SC on day 4 of each chemotherapy cycle, approximately 24 h after the last chemotherapy infusion and after blood sampling to determine ANC and body temperature. The lipegfilgrastim 6-mg dose was chosen based on findings from a phase II dose-finding study in breast cancer patients that demonstrated neutrophil support that was at least equivalent to the standard 6.0-mg fixed dose of pegfilgrastim. Patients who developed FN in any cycle were not discontinued except by investigator decision; instead, they received open-label prophylactic treatment with lipegfilgrastim in subsequent cycles, regardless of treatment group.

### Efficacy assessments

The primary efficacy measure was the incidence of FN in cycle 1. Febrile neutropenia was defined as an oral body temperature >38.5°C for at least 1 h (two consecutive same-day measurements, ≥60 min apart) with severe neutropenia (ANC <0.5 × 10^9^/L) on the day before, same day, or day after the elevated temperature readings; neutropenic sepsis (sepsis with ANC <0.5 × 10^9^/L); or serious or life-threatening neutropenic infection (infection with ANC <0.5 × 10^9^/L).

Secondary efficacy measures included the incidence of FN in cycles 2, 3, and 4 and across all cycles; incidence and DSN (defined as grade 4 neutropenia with ANC <0.5 × 10^9^/L); incidence and duration of very severe neutropenia (ANC <0.1 × 10^9^/L); depth of ANC nadir (lowest ANC in each cycle); time to ANC nadir (defined as time from chemotherapy administration until occurrence of ANC nadir); time to ANC recovery (defined as time from chemotherapy administration until ANC increased to ≥2.0 × 10^9^/L after nadir); and time to ANC recovery from ANC nadir (defined as the difference in days between day of ANC nadir to first day with ANC ≥1.5 × 10^9^/L).

Blood samples to determine ANC were obtained daily until day 15, or longer, of each cycle, until ANC ≥2.0 × 10^9^/L was reached. A blood sample was taken on day 4 of each cycle, before administration of study medication. Body temperature was measured orally at least twice daily (morning and evening) until day 15, or longer, of each cycle, until ANC ≥2.0 × 10^9^/L was reached.

Other secondary efficacy measures, including hospitalizations, use of IV antibiotics, delivered versus scheduled chemotherapy, chemotherapy dose modifications (reductions, omissions, delays), quality of life, and the incidence of patients requiring prophylactic open-label treatment, as well as pharmacokinetic properties were assessed but are not reported here.

### Safety assessments

Safety was assessed using reported treatment-emergent AEs data, including intercurrent illnesses and clinically abnormal laboratory values; AEs were recorded until 3 weeks after the last injection of study medication. Adverse events were summarized by seriousness, severity, and investigator-assessed relationship to study medication. A serious AE was one that was life-threatening or resulted in death, required hospitalization or prolongation of hospitalization, or resulted in a persistent or significant disability or incapacity that required medical or surgical intervention. Investigators assessed AEs as probably, possibly, unlikely, or not related to the study medication or chemotherapy regimen, or as not classifiable. For laboratory values, all AEs of grade 3 and higher were documented. The AEs were assessed on days 1 and 7 of each chemotherapy cycle. Safety samples (hematology and clinical chemistry) were taken on day 15. Other safety assessments, including physical examination and vital signs, were performed within 24 h before chemotherapy administration on day 1 and on days 7 and 15 of each chemotherapy cycle.

### Statistical analysis

The planned sample size was 375 patients from approximately 90 centers in nine countries, based on the assumption that the incidence of FN would be in the range of 7% to 10% in the placebo group and, at most, 1% in the lipegfilgrastim group. For a statistical test with a two-sided significance level α of 5%, a required power of at least 80%, and a sampling rate of 2:1 (lipegfilgrastim:placebo), sample size requirements were 250 patients in the lipegfilgrastim group and 125 patients in the placebo group. As the actual incidence of FN in the placebo group was expected to be closer to 10%, a power of at least 90% was expected.

For the primary efficacy measure (incidence of FN in cycle 1), a 95% CI for the OR (placebo/lipegfilgrastim) was calculated to assess the relative efficacy of lipegfilgrastim versus placebo. For secondary efficacy measures, no adjustment for Type I error was applied, so all secondary analyses should be interpreted as exploratory. When applicable, for secondary efficacy measures for which regression analyses were planned, statistical models with the same explanatory variables as in the analysis of the primary measures were estimated. Demographic and baseline characteristics, AEs, and other safety assessments were presented as descriptive statistics (continuous variables) or frequency tables (categorical variables).

The intent-to-treat population, which included all patients randomized at baseline, was also the efficacy population. The safety population included all randomized patients who received at least one dose or partial dose of study medication.

## Endnote

Results from this study were presented in part at the Multinational Association of Supportive Care in Cancer International Symposium, New York, NY, June 28–30, 2012.

## Abbreviations

AEs: adverse events
ANC: absolute neutrophil count
CI: confidence interval
DSN: duration of severe neutropenia
ECOG PS: Eastern Cooperative Group Performance Status
FN: febrile neutropenia
G-CSFs: granulocyte colony-stimulating factors
IV: intravenous
NSCLC: non-small cell lung cancer
PEG: polyethylene glycol
SC: subcutaneous
